# Potential Interaction of Pinocembrin with Drug Transporters and Hepatic Drug-Metabolizing Enzymes

**DOI:** 10.3390/ph18010042

**Published:** 2025-01-01

**Authors:** Sirima Sangkapat, Rattiporn Boonnop, Jeerawat Pimta, Napason Chabang, Bodee Nutho, Promsuk Jutabha, Sunhapas Soodvilai

**Affiliations:** 1 Department of Pharmaceutical Technology, College of Pharmacy, Rangsit University, Pathumthani 12000, Thailand; 2 Research Center of Transport Protein for Medical Innovation, Department of Physiology, Faculty of Science, Mahidol University, Ratchathewi, Bangkok 10400, Thailand; rattiporn.rb@gmail.com (R.B.); jeerawat.pimta@gmail.com (J.P.); 3 School of Bioinnovation and Bio-based Product Intelligence (SCIN), Faculty of Science, Mahidol University, Ratchathewi, Bangkok 10400, Thailand; napason.cha@mahidol.ac.th; 4 Department of Pharmacology, Faculty of Science, Mahidol University, Ratchathewi, Bangkok 10400, Thailand; bodee.nut@mahidol.ac.th; 5 Chakri Naruebodindra Medical Institute, Faculty of Medicine Ramathibodi Hospital, Mahidol University, Bang Phli, Samut Prakan 10540, Thailand; promsuk.jut@mahidol.ac.th; 6 Excellent Center for Drug Discovery, Mahidol University, Ratchathewi, Bangkok 10400, Thailand

**Keywords:** OAT1, OAT3, CYP2C19, flavonoids, drug interaction, drug development

## Abstract

**Background/Objectives**: Pinocembrin is a promising drug candidate for treating ischemic stroke. The interaction of pinocembrin with drug transporters and drug-metabolizing enzymes is not fully revealed. The present study aims to evaluate the interaction potential of pinocembrin with cytochrome P450 (CYP450: CYP2B6, CYP2C9, and CYP2C19) and drug transporters including organic anion transporters (OAT1 and OAT3), organic cation transporters (OCT1 and OCT2), multidrug and toxin extrusion (MATE1 and MATE2, P-glycoprotein (P-gp), and breast cancer resistance protein (BCRP). **Methods**: The interactions of pinocembrin on drug transporters were determined in the Madin–Darby canine kidney (MDCK) cells overexpressing human (h)OAT1 or hOAT3 and in the Chinese hamster ovary (CHO-K1) cells overexpressing hOCT1, hOCT2, hMATE1, or hMATE2. The interactions of pinocembrin with BCRP and P-glycoprotein were determined in Caco-2 cells. The CYP450 enzyme inhibitory activity was assessed by a cell-free CYP450 screening assay. **Results**: Pinocembrin effectively inhibited the function of OAT1 and OAT3 with a half-inhibitory concentration (IC_50_) and inhibitory constant (Ki) of ∼2 μM. In addition, it attenuated the toxicity of tenofovir, a substrate of hOAT1, in cells overexpressing hOAT1. Based on the kinetic study and molecular docking, pinocembrin inhibited OAT1 and OAT3 via a competitive inhibition. In contrast to hOAT1 and hOAT3, pinocembrin did not significantly inhibit the function of OCT1, OCT2, MATE1, MATE2, BCRP, and P-glycoprotein. In addition, pinocembrin potently inhibited the activity of CYP2C19, whereas it exhibited low inhibitory potency on CYP2B6 and CYP2C9. **Conclusions**: The present study reveals the potential drug interaction of pinocembrin on OAT1, OAT3, and CYP2C19. Co-administration with pinocembrin might affect OAT1-, OAT3-, and CYP2C19-mediated drug pharmacokinetic profiles.

## 1. Introduction

Pinocembrin is a bioactive compound in a group of flavonoids. It is isolated from various natural sources, such as honey, propolis, finger root, wild marjoram, piper leaves, oregano, licorice aerial parts, and other plants [1]. Pinocembrin exhibits several pharmacological effects, such as anti-oxidant [2], anti-microbial [3], anti-inflammatory [4], anti-cancer [5], cardioprotective [6], and neuroprotective activities [7]. Specifically, it is well known as a potential neuroprotective agent for ischemic stroke [8]. Pinocembrin can protect rats from cerebral ischemia and improve neurological function by multiple mechanisms, including mitochondrial protection, the inhibition of autophagy, antioxidation, anti-apoptosis, and anti-autophagy [9]. Given its significant efficacy in ischemic stroke, pinocembrin was a promising drug candidate for progressing to a phase II clinical trial in China (NCT02059785). In this randomized, double-masked, placebo-controlled, multicenter study, the patients were treated with 40 mg and 60 mg pinocembrin injections twice daily for 14 days [10]. The pharmacokinetic of pinocembrin at a dose of 80 mg intravenous injection in human subjects revealed that the maximal plasma concentration (Cmax) and the time required for the plasma concentration of a drug to decrease by 50% (T1/2) were 1.22 μg/mL and 55 min, respectively [10]. Although the therapeutic effects of pinocembrin are extensive, the information concerning drug interaction requires further investigation.

The key mechanisms responsible for pharmacokinetic interactions are the activity of drug transporters. Several drug transporters play crucial roles in therapeutic drug interactions, for example, breast cancer resistance protein (BCRP), P-glycoprotein (P-gp), organic anion transporters (OATs), organic cation transporters (OCTs), organic anion transporting polypeptides (OATPs), and multidrug and toxin extrusion (MATE). In addition to drug transporters, drug-metabolizing enzymes, especially cytochrome P450 (CYP450), also contribute to drug interactions [11,12,13]. The alteration of these drug transporters and CYP450 leads to changes in drug pharmacokinetic profiles. Previous studies have demonstrated that pinocembrin inhibits the function of OATP2B1 and OATP1A2 in transfected renal cells. Pinocembrin inhibits OATP2B1-mediated rosuvastatin uptake with a half-inhibitory concentration (IC_50_) of 8.9 µM, whereas it weakly inhibits OATP1A2 function [14]. The pharmacokinetic data of pinocembrin reveal that the maximal plasma concentration of pinocembrin following intravenous injections of pinocembrin of 40 mg and 80 mg are 0.6 µg/mL (2.3 µM) and 1.22 µg/mL (4.7 µM), respectively, which do not reach the IC_50_ values for OATP2B1-mediated statin drug transport [10]. In addition to OATP2B1 and OATP1A2, pinocembrin did not inhibit the function of P-gp, whereas it weakly inhibited BCRP [15,16]. However, the information concerning the effect of pinocembrin on these endogenously expressed transporters in intestinal cells is lacking. In addition to the drug transporters, pinocembrin, with concentrations of 1–5 µM, shows an inhibitory effect on the activity of CYP3A4 and CYP1A2. However, it does not affect the activity of CYP2A6 and CYP2C8 [17,18,19]. Even though previous studies have studied the effect of pinocembrin on some drug transporters and drug-metabolizing enzymes, a comprehensive investigation of pinocembrin interactions with transporters and enzymes requires immediate attention.

Since pinocembrin has been developed as a new drug for the treatment of ischemic stroke, it is essential to investigate whether pinocembrin has the potential to cause drug interaction. The present study reveals whether pinocembrin alters drug transporters and the CYP450 enzymes responsible for the pharmacokinetics of therapeutic drugs. The interactions of pinocembrin with drug transporters such as OAT1, OAT3, OCT1, OCT2, MATE1, MATE2, P-gp, and BCRP, and drug-metabolizing enzymes, including CYP2B6, CYP2C9, and CYP2C19, are investigated in this study.

## 2. Results

### 2.1. The Effect of Pinocembrin on the Function of Drug Transporters

**Figure 1:**
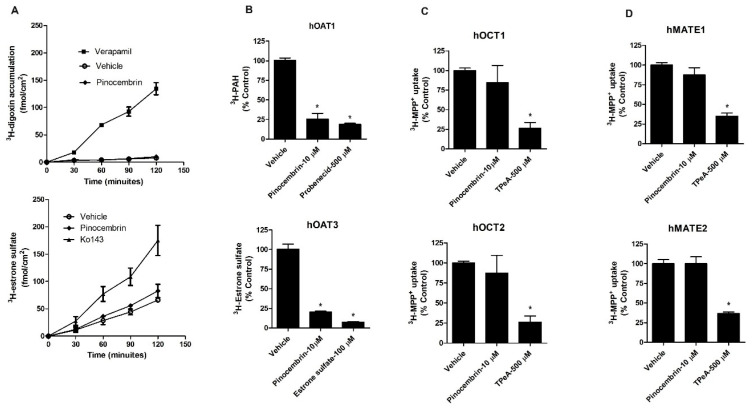
Effect of pinocembrin on the function of P-gp, BCRP, hOAT1, hOAT3, hOCT1, hOCT2, hMATE1, and hMATE2. (**A**) Transport function of P-gp and BCRP, (**B**) hOAT1 and hOAT3, (**C**) hOCT1 and hOCT2, and (**D**) hMATE1 and hMATE3. Probenecid and estrone sulfate were positive controls for hOAT1 and hOAT3 inhibition. Tetraethylammonium (TPeA) is an inhibitor of hOCT1, hOCT2, hMATE1, and hMATE2. The data are expressed as the mean ± S.D. of % of vehicle control from three experiments. * *p* < 0.05 compared with the control.

The interactions of pinocembrin with P-gp and BCRP were determined by the measurement of P-gp-mediated ^3^H-digoxin transport and BCRP-mediated ^3^H-estrone sulfate transport in Caco-2 cell monolayers, which express both P-gp and BCRP [20]. The apical sides of the Caco-2 cell monolayers were incubated with a buffer containing ^3^H-digoxin alone, ^3^H-digoxin plus 10 µM pinocembrin or ^3^H-digoxin plus 100 µM verapamil (P-gp inhibitor). The accumulation of ^3^H-digoxin at the basolateral side was determined at 0, 30, 60, 90, and 120 min. The results showed that verapamil increased the transport of ^3^H-digoxin from the apical side to the basolateral side compared with the vehicle control, indicating P-gp inhibition. Pinocembrin did not alter the transport of ^3^H-digoxin from the apical to basolateral side compared with the vehicle control. In addition, the inhibition of BCRP by Ko143 increased the transport of ^3^H-estrone sulfate from the apical to basolateral side compared with the vehicle control. Like P-gp, pinocembrin did not affect ^3^H-estrone sulfate transport (Figure 1A). These results indicate that pinocembrin did not significantly affect P-gp- and BCRP-mediated transepithelial transport.

Next, the effects of pinocembrin on the transport function of renal drug transporters, including hOAT1, hOAT3, hOCT1, hOCT2, hMATE1, and hMATE2, were determined. Pinocembrin (10 µM) significantly decreased ^3^H-aminohippuric acid (^3^H-PAH), a substrate of hOAT1, and ^3^H-estrone sulfate, a substrate of hOAT3, cellular accumulation compared with the vehicle control in MDCK cells overexpressing hOAT1 and hOAT3, respectively (Figure 1B). Non-radiolabeled probenecid and estrone sulfate were used to competitively inhibit the function of hOAT1 and hOAT3, respectively. The data indicate that pinocembrin inhibits the transport function of hOAT1 and hOAT3. In contrast to hOAT1 and hOAT3, pinocembrin did not alter the hOCT1-, hOCT2-, hMATE1-, and hMATE2-mediated uptake of ^3^H-MPP^+^, which is a prototypic substrate of these transporters (Figure 1C,D). The transport of hOAT1, hOAT3, hOCT1, hOCT2, hMATE1, and hMATE2 substrates in mock and non-transfected parent cells showed no significant difference. These data support the idea that pinocembrin does not alter the function of hOCT1, hOCT2, hMATE1, and hMATE2.

### 2.2. Inhibitory Potency and the Mechanism of Pinocembrin Inhibition on hOAT1 and hOAT3

**Figure 2:**
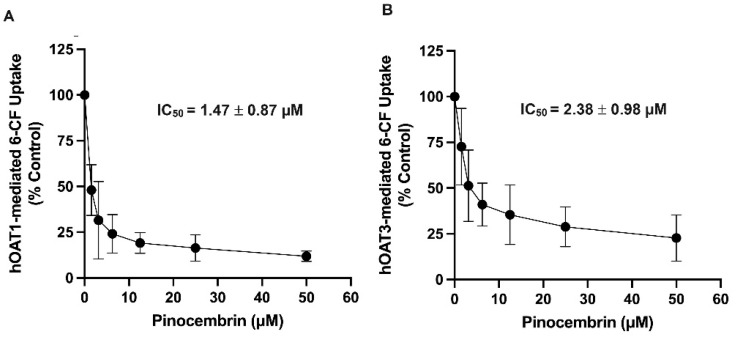
Inhibitory potency of pinocembrin on the transport function of hOAT1 and hOAT3. (**A**) hOAT1-MDCK cells and (**B**) hOAT3-MDCK cells incubated with 6-CF (10 µM) in the presence of pinocembrin for 20 min. Uptakes of 6-CF are calculated as % of control (no pinocembrin) and represented as the mean ± S.D. from three experiments.

**Figure 3:**
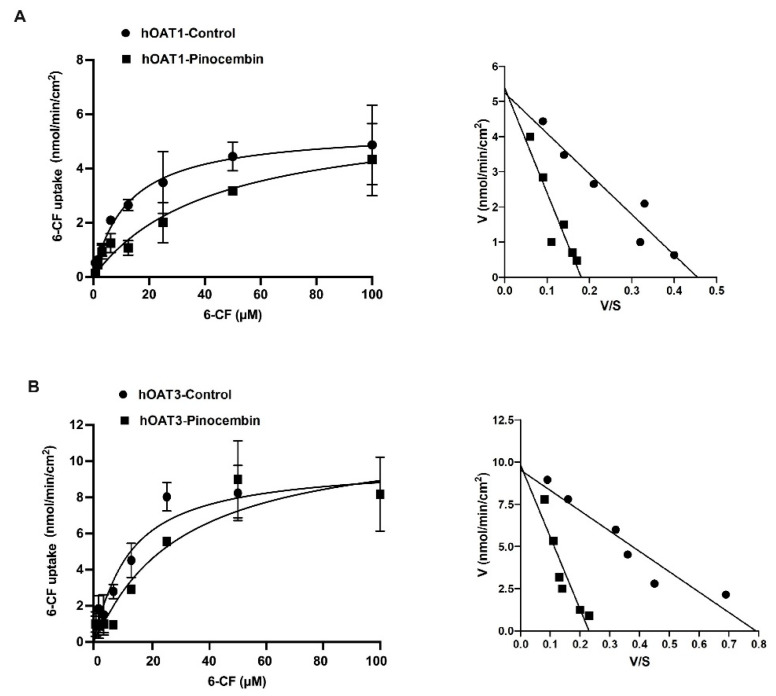
Kinetic studies on hOAT1- and hOAT3-mediated 6-CF uptake. The uptake of 6-CF (1–100 μM) for 20 min in hOAT1-MDCK cells (**A**) and hOAT3-MDCK cells (**B**) in the presence of the vehicle and 5 μM pinocembrin. The Michaelis–Menten diagrams and Eadie–Hofstee plots of 6-CF uptake are shown.

To determine the inhibitory potency of pinocembrin on the hOAT1 and hOAT3 function, the IC_50_ of pinocembrin on the hOAT1- and hOAT3-mediated uptake of 6-carboxyfluorescein (6-CF), a fluorescent substrate of hOAT1 and hOAT3, was determined. As shown in Figure 2, pinocembrin inhibited hOAT1- and hOAT3-mediated 6-CF transport in a concentration-dependent manner. The IC_50_ values and the Ki of pinocembrin were approximately 1–3 µM, as shown in Table 1. Next, the inhibitory mechanism of pinocembrin was determined using a kinetic study. The kinetic parameters showed that in the presence of 5 μM pinocembrin, the Michaelis–Menten constant (K_m_) of hOAT1 and hOAT3 significantly increased. In contrast, the maximal velocity (V_max_) did not change substantially compared with the vehicle control (Table 1 and Figure 3). These results support the theory that pinocembrin may competitively inhibit the transport function of hOAT1 and hOAT3.

### 2.3. Molecular Docking of Pinocembrin with hOAT1 and hOAT3

**Figure 4:**
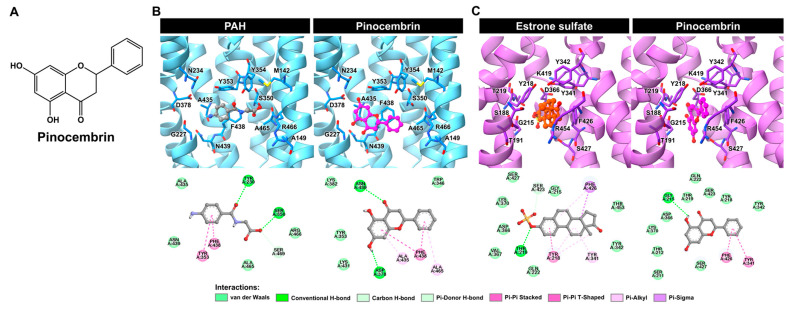
Binding prediction of pinocembrin with hOAT1 and hOAT3. (**A**) Chemical structure of pinocembrin. (**B**,**C**) The molecular docking results of pinocembrin, in complex with hOAT1 and hOAT3, represented as 3D close-up views of the substrate-/inhibitor-binding site and 2D interaction diagrams of ligand–hOAT1/hOAT3 complexes.

Molecular docking was performed to predict the binding modes of pinocembrin when it inhibits hOAT1 and hOAT3. As illustrated in Figure 4A, the docking results revealed that pinocembrin occupied the same binding site as the substrate molecules (i.e., aminohippuric acid (PAH) for hOAT1 and estrone sulfate for hOAT3). This suggests that pinocembrin may act as a competitive binding agent of the transporters. Further insights from 2D interaction diagrams revealed that PAH primarily interacted with hOAT1 through conventional hydrogen bonds (H-bonds) involving Y230 and S350, π-π T-shaped the interaction with Y353, π-π stacking with F438, and non-classical carbon H-bonding with S469. It is worth noting that the binding orientation of PAH in the hOAT1 binding site obtained from the docking simulation closely resembled the cryo-EM structure of rat OAT1 in a complex with PAH [21].

Moreover, it was observed that pinocembrin could interact with additional residues at the substrate-binding site of hOAT1 and form various types of interactions. These results implied the potent inhibition of hOAT1. In the hOAT3–ligand complex, the residues of hOAT3 mainly interacted with its substrate, estrone sulfate, through π-π stacking (Y218), π-alkyl (Y218, Y341, and F426), and π-sigma (F426), as shown in Figure 4B. While these π-related interactions played a minor role in the binding of pinocembrin with hOAT3, van der Waals interactions were more important in that regard. In fact, hOAT3 prefers compounds with a higher molecular weight and greater lipophilicity than hOAT1 [22]. Consistent with the docking study, pinocembrin, a relatively hydrophobic molecule, could embed itself in the substrate-binding site of hOAT3 and potentially inhibit hOAT3 activity. Thus, docking simulations, in part, shed light on the mode of action of pinocembrin against hOAT1 and hOAT3.

### 2.4. The Effect of Pinocembrin Treatment on the hOAT1 and hOAT3 Function

**Figure 5:**
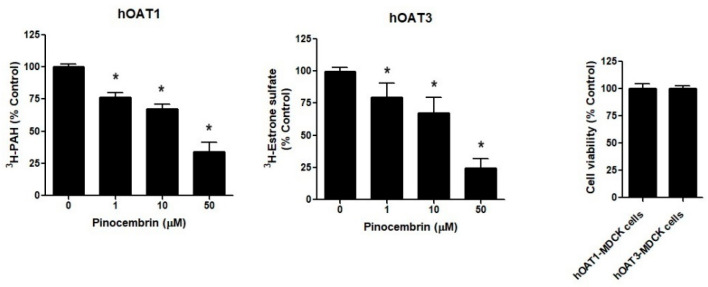
The effect of pinocembrin treatment on hOAT1 and hOAT3. The hOAT1-MDCK and hOAT3-MDCK cells were incubated with pinocembrin for 48 h, followed by the measurement of the hOAT1 and hOAT3 transport function and cell viability. The data are the mean ± S.D. of three experiments. * *p* < 0.05 compared with control.

The hOAT1-MDCK cells and hOAT3-MDCK cells were treated with 1, 10, and 50 μM pinocembrin for 24 h, followed by a measurement of ^3^H-PAH and ^3^H-estrone sulfate cellular accumulation. As shown in Figure 5, pinocembrin significantly decreased the ^3^H-PAH and ^3^H-estrone sulfate accumulation in hOAT1-MDCK and hOAT3-MDCK cells, respectively, compared with the vehicle treatment. The inhibitory effect of pinocembrin occurred in a concentration-dependent manner. The cells incubated with pinocembrin did not affect cell viability compared to the vehicle-treated cells, indicating that the inhibitory effect of pinocembrin did not result from pinocembrin-induced toxicity.

### 2.5. The Effect of Pinocembrin on Tenofovir Induces Toxicity in hOAT1-MDCK Cells and hOAT3-MDCK Cells

**Figure 6:**
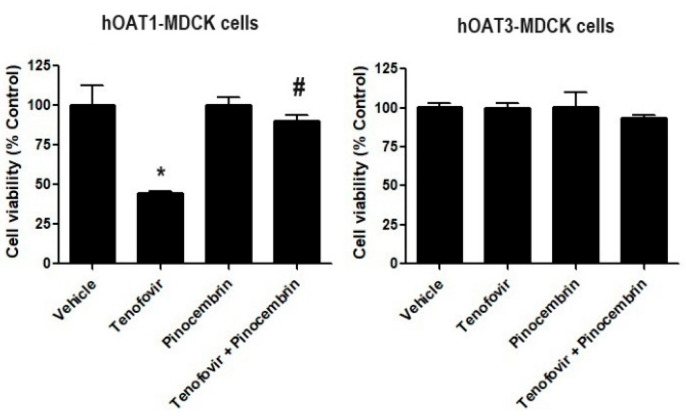
The effect of pinocembrin treatment on hOAT1 and hOAT3. The hOAT1-MDCK and hOAT3-MDCK cells were incubated with indicated conditions for 48 h, then the viability of hOAT1-MDCK and hOAT3-MDCK cells was measured. Data are the mean ± S.D. of three experiments. * *p* < 0.05 compared with vehicle and ^#^ *p* < 0.05 compared with tenofovir-treated cells.

We determined whether the inhibitory effect of pinocembrin on hOAT1 and hOAT3. We studied whether pinocembrin could reduce the renal toxicity of tenofovir, a substrate of hOAT1 and hOAT3 known for inducing renal tubular dysfunction [23]. The results revealed that tenofovir at 5 µM significantly reduced the cell viability of hOAT1-MDCK cells, whereas it did not affect the viability of hOAT3-MDCK cells. The co-treatment of hOAT1-MDCK cells with tenofovir and pinocembrin considerably increased cell viability compared with tenofovir-treated cells alone (Figure 6).

### 2.6. The Effect of Pinocembrin on the Activity of CYP Enzymes

**Figure 7:**
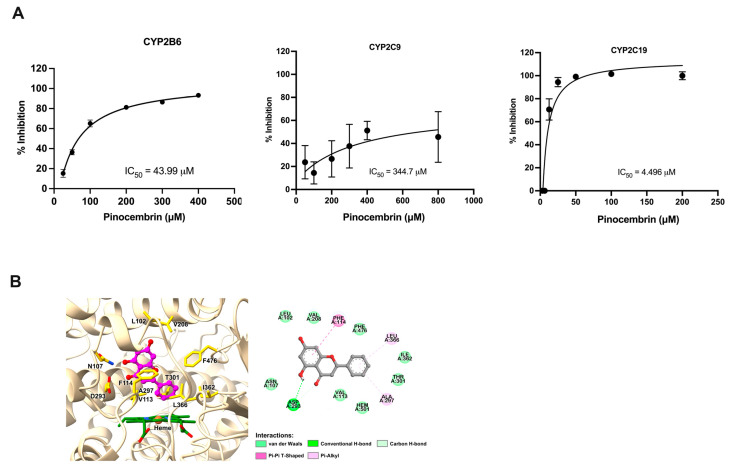
Effects of pinocembrin on the activity of CYP enzymes. (**A**) Inhibitory effect of pinocembrin on CYP2B6, CYP2C9, and CYP2C19. (**B**) Molecular docking result of pinocembrin in complex with human CYP2C19 (3D close-up view of active site of CYP2C19 and 2D interaction diagram of pinocembrin–CYP2C19 complex).

To determine whether pinocembrin influences the catalytic activity of CYP450 enzymes, fluorescent probe reaction assays were performed using varying concentrations of pinocembrin. As presented in Figure 7A, the enzyme inhibition study showed that pinocembrin strongly inhibited CYP2C19 with IC_50_ values of 4.49 μM. Our data revealed that pinocembrin had a low inhibitory potency on CYP2B6 and CYP2C9 with IC_50_ values of 43.99 μM and 344.70 μM, respectively. In addition, molecular docking was conducted to better understand the binding mode and interactions between pinocembrin and human CYP2C19. As shown in Figure 7B, the docking analysis revealed that pinocembrin interacted with multiple amino acid residues in the enzyme’s active site. Several residues of CYP2C19, including L102, N107, V113, F114, V208, D293, A297, T301, I362, L366, and F476, played important roles in forming stable interactions with pinocembrin. Specifically, A297 and L366 established crucial contacts with the phenyl ring (B ring) of pinocembrin through π-alkyl interactions. Additionally, D293 engaged in a conventional H-bond and a non-classical carbon H-bond with the hydroxyl group on the benzyl moiety (A ring) and the carbonyl group on the C ring of pinocembrin, respectively. A π-π T-shaped interaction was also formed between the F114 residue and the A ring of pinocembrin.

## 3. Discussion

Pinocembrin, a bioactive compound found in several herbs, has demonstrated promising prospects in treating neurological diseases through its involvement in numerous pharmacological activities [1]. Pinocembrin did not inhibit the transport of prototypical substrates P-gp, BCRP, hOCT1, hOCT2, hMATE1, and hMATE2, indicating that it might not alter the transport function of these transporters, and, in turn, the pharmacokinetics of the drugs transported by these transporters. The data on P-gp were similar to the previous study in that pinocembrin had little effect on the P-gp in the blood–brain barrier (BBB). Pinocembrin may not affect the functional activity and protein expression of the P-gp transporter at the BBB [15].

Pinocembrin showed a high potency in inhibiting hOAT1 and hOAT3, which play a crucial role in the renal clearance of anionic therapeutic drugs [24]. As the data on the kinetic parameter and molecular docking supports the idea that pinocembrin inhibits hOAT1-mediated PAH uptake and hOAT3-mediated estrone sulfate uptake, the drug transporter inhibition mode could be a competitive mechanism. However, it is uncertain whether pinocembrin is a substrate of hOAT1 and hOAT3 or if it acts as an inhibitor of hOAT1 and hOAT3. Since pinocembrin demonstrates an inhibitory effect on hOAT1 and hOAT3, we determined whether its inhibition affected drug transport into the cells. The present study reveals that pinocembrin could attenuate the tenofovir-induced toxicity of hOAT1-MDCK cells. hOAT1 is the main transporter responsible for tenofovir uptake into renal proximal tubular cells [23], implying that pinocembrin inhibits tenofovir uptake into the cells. Since pinocembrin inhibits OATP2B1 and OATP1A2, affecting statin uptake [14], it can be implied that pinocembrin may selectively affect the transport of organic anionic drugs rather than organic cationic drugs.

It is important to reveal that the inhibitory effect of pinocembrin on hOAT1 and hOAT3 has clinical relevance to drug interaction. The IC_50_ of pinocembrin on hOAT1 and hOAT3 is approximately 2 µM, falling within the observed plasma concentration of the study on phase I clinical trials for the pharmacokinetic investigation of pinocembrin (approximately 3–10 µM) [10]. Based on the current criteria used by the U.S. FDA, a clinically relevant drug interaction is of interest when the unbound Cmax/IC_50_ values are ≥0.1 [11]. Protein binding data reveal that approximately 60% of pinocembrin binds to serum proteins. These data indicate that the unbound Cmax might affect the OAT1/3 function. Since OAT1 and OAT3 play a crucial role in renal clearance of several groups of therapeutic drugs such as β-lactam antibiotics (penicillin and cephalosporins), antiviral agents (acyclovir, adefovir, didanosine, lamivudine, stavudine, zalcitabine, and zidovudine), diuretics (bumetanide and furosemide), NSAIDs (indomethacin, salicylate, ibuprofen, and ketoprofen) [12], coadministration of pinocembrin and these groups of drugs may render clinically relevant. The clinical relevance of pinocembrin on narrow therapeutic drugs should be further investigated.

There are more than 50 CYP450 enzymes, the most significant being CYP1A2, CYP2C9, CYP2C19, CYP2D6, CYP3A4, CYP3A5, CYP2D6, and CYP2E1, which metabolize 90 percent of drugs [13]. The inhibition and induction of CYP450 enzymes are the major mechanisms causing pharmacokinetic interaction. Previous studies have demonstrated that pinocembrin inhibits the activities of CYP2D6 and CYP3A4 [17,18,25]. However, there is limited literary evidence regarding its inhibitory effect on other CYP450s involved in drug metabolism. The findings of this study reveal that pinocembrin acts as a potent inhibitor of CYP2C19 and weakly inhibits CYP2B6 and CYP2C9. There are various types of CYP450 reversible inhibition (competitive and non-competitive) and irreversible (mechanism-based) inhibition [26]. A study by Bhatt S. et al. [19] revealed that pinocembrin is a potential inhibitor of CYP1A2, with the mechanism of inhibition identified as competitive. It is uncertain whether the mechanism of pinocembrin inhibition on CYP2C19 is the same as that on CYP1A2. The mechanism behind pinocembrin’s inhibition of CYP2C19 requires further study. CYP2C19 plays a role in the clearance of several drugs, such as oral anticoagulants, chemotherapeutic agents, anti-epileptics, antiplatelets (clopidogrel), proton pump inhibitors, and antidepressants [27]. Therefore, administering pinocembrin may alter the CYP2C19-mediated hepatic clearance of drugs. A limitation of this study is that all data are obtained from in vitro experiments; thus, in terms of the overall outcome of the interaction, it is uncertain whether pinocembrin inhibits or stimulates the CYP2C19 function. As previous studies and our data reveal that pinocembrin inhibits CYP3A4, CYP1A2, and CYP2C19, drugs that are metabolized by these enzymes might be affected by pinocembrin administration. The potential risk of pinocembrin on CYP3A4-, CYP1A2-, and CYP2C19-mediated drug interaction must be further verified through an in vivo study.

## 4. Materials and Methods

### 4.1. Materials

The ^3^H-digoxin (23.8 Ci/mmol), ^3^H-aminohippuric acid (^3^H-PAH; 5.0 Ci/mmol), ^3^H-estrone sulfate (55.4 Ci/mmol), and ^3^H-methyl-4-phenylpyridinium (^3^H-MPP^+^; 82.9 Ci/mmol) were purchased from PerkinElmer (Bangkok, Thailand). The pinocembrin, verapamil hydrochloride, probenecid, estrone sulfate, tetraethylammonium (TPeA), 6-CF, tenofovir, and 3-(4,5-Dimethylthiazol-2-yl)-2,5-Diphenyltetrazolium Bromide (MTT) were purchased from Sigma-Aldrich (St. Louis, MO, USA). The Ko143 was purchased from MedChem Express (Monmouth Junction, NJ, USA). The culture media, fetal bovine serum (FBS), G418, L-glutamine, non-essential amino acids, penicillin and streptomycin, and trypsin–EDTA solution were obtained from Gibco Life Technology (Grand Island, NY, USA).

### 4.2. Cell Culture

Human colon adenocarcinoma (Caco-2) cells obtained from ATCC (Manassas, VA, USA) were cultured in Dulbecco’s Modified Eagle Medium (DMEM), high-glucose supplemented with 20% FBS, 4 mM L-glutamine, and 20 mM non-essential amino acids, 100 U/mL penicillin, and 100 μg/mL streptomycin. Madin–Darby canine kidney (MDCK) cells obtained from AddexBio Technologies (San Diego, CA, USA) were maintained in minimum essential medium (MEM) supplemented with 10% FBS, 100 U/mL penicillin, and 100 μg/mL streptomycin. To generate MDCK cells overexpressing hOAT1 (hOAT1-MDCK) and hOAT3 (OAT3-MDCK), the parent MDCK cells were transfected with the expression plasmids of hOAT1 (# SC303532) or hOAT3 (# SC122673) or an empty pCMV6 vector (OriGene Technologies, Inc., Rockville, MD, USA). After transfection, the hOAT1-MDCK and hOAT3-MDCK cells were selected by complete MEM containing 700 µg/mL G418 for eight weeks. The Chinese hamster ovary (CHO-K1) cells obtained from CLS (Deutschland, Germany) were cultured in complete Kaighn’s modification of Ham’s F-12 (with 10% FBS, 100 U/mL penicillin, and 100 μg/mL streptomycin). The CHO-K1 cells overexpressing hOCT1, hOCT2, hMATE1, and hMATE2 were generated by the transfection of parent CHO-K1 cells with the expression plasmids of hOCT1 (#RC211872), hOCT2 (#RC207921), hMATE1 (#RC201890), and hMATE2 (#RC223318) or an empty pCMV6 vector (OriGene Technologies, Inc., Rockville, MD, USA). Transporter-overexpressing cells were selected with 3 mg/mL of G418. Cells were cultured at 37 °C in a humidified atmosphere containing 5% CO_2_.

### 4.3. Transepithelial Transport Assay

P-gp- and BCRP-mediated transport were measured by the transepithelial transport of their substrates (^3^H-digoxin for P-gp and ^3^H-estrone sulfate for BCRP) in Caco-2 cell monolayers. This experiment was performed as previously described [24,25]. Caco-2 cells were cultured on membrane inserts (Corning Inc., Corning, NY, USA) for 21 days. Cell monolayers showing transepithelial electrical resistance values > 600 Ω.cm^2^ were included in the experiment. On the day of the experiment, the monolayers were washed three times with a warm transport buffer (37 °C) containing 135 mM NaCl, 5 mm KCl, 1.2 mM MgCl_2_, 2.5 mM CaCl_2_, 0.8 mM MgSO_4_, and 28 mM D-glucose, 13 mM HEPES. The cell monolayers were further incubated with transport buffer for 15 min at 37 °C. The apical side of the cell monolayer was incubated with a transport buffer containing the radioactive substrate (^3^H-digoxin or ^3^H-estrone sulfate) alone or with pinocembrin or the inhibitor of the drug transporters (verapamil for P-gp and Ko143 for BCRP). The basolateral side was replaced with a transport buffer. The sample from the basolateral side was transferred to vials containing a liquid scintillation cocktail to measure radioactivity by a β-counter (Packard BioScience, Wellesley, MA, USA).

### 4.4. Cis-Inhibition Assay

A cell monolayer comprising hOAT1-MDCK, hOAT3-MDCK, hOCT1-CHO-K1, hOCT2-CHO-K1, hMATE1-CHO-K1, hMATE2-CHO-K1, and mock cells were utilized for the uptake experiments. The cells were washed three times with a buffer containing (in mM) 140 NaCl, 5 KCl, 0.4 MgSO_4_, 0.5 MgCl_2_, 1 CaCl_2_, 0.3 Na_2_HPO_4_, 0.4 KH_2_PO_4_, 6 D-glucose, and 4 NaHCO_3_ at pH 7.4 and incubated at 37 °C for 15 min. After incubation, the hOAT1-MDCK and hOAT3-MDCK cells were incubated for 10 min with ^3^H-PAH and ^3^H-estrone sulfate, a substrate of hOAT1 and hOAT3, respectively. The hOCT1-CHO-K1 and hOCT2-CHO-K1 cells were incubated with ^3^H-MPP^+^, a substrate of OCT1 and OCT2, for five minutes. The functions of MATE1 and MATE2 were determined by incubating the hMATE1-CHO-K1 and hMATE2-CHO-K1 cells with a buffer containing ^3^H-MPP^+^, a substrate of MATE1 and MATE2, at pH 8.5 for five minutes. The cells were washed three times with an ice-cold buffer to stop the transport function. The cells were lyzed with 0.4 N NaOH for at least four hours. The samples were collected and transferred to vials containing a liquid scintillation cocktail to measure radioactivity by a β-counter (Packard BioScience, Wellesley, MA, USA). The cellular accumulation of radioactive substrates was shown as the percentage of control.

### 4.5. Determining the Inhibitory Potency of Pinocembrin on OATs-Mediated Transport

The hOAT1-MDCK and hOAT3-MDCK cell monolayers were incubated at 37 °C with an uptake buffer containing 6-CF (10 µM), a fluorescent substrate of hOAT1 and hOAT3 [12], and pinocembrin (0–50 µM) for 10 min. Uptake was terminated by removing the uptake solution and washing four times with a cold buffer. The cell monolayers were lysed with 0.4 N NaOH for 30 min. The samples were transferred to 96-black well plates to measure the fluorescence intensity using a fluorescent plate reader (EnVision XCite 2105 Multimode Plate Reader, Packard BioScience, Wellesley, MA, USA) at excitation and emission wavelengths of 490 and 510, respectively. The IC_50_ of pinocembrin on hOAT1- and hOAT3-mediated 6-CF uptake was calculated in GraphPad Prism using sigmoidal concentration–response analysis using the equation Y = 100/(IC_50_/X)^HillSlope^ (X is the concentration, while Y is the normalized response). In addition, the estimated inhibitory constant (Ki) was calculated using the Cheng–Prusoff equation.

### 4.6. Kinetics of hOAT-Mediated Transport

The hOAT1-MDCK and hOAT3-MDCK cell monolayers were incubated with 6-CF at increasing concentrations (0 to 100 µM) and in the absence or presence of 5 µM pinocembrin for 10 min. The kinetic parameters of 6-CF transport by hOAT1 and hOAT3 in the presence of pinocembrin were determined using nonlinear regression analysis to fit the Michaelis–Menten kinetic equation:

$$V=V_{\max}\frac{\left[S\right]}{K_{m}+\left[S\right]}$$

where V is the rate of 6-CF transport, V_max_ is the maximum rate of 6-CF transport, K_m_ is the 6-CF concentration that results in half the maximum transport (Michaelis–Menten constant), and [S] is the concentration of 6-CF in the transport reaction.

### 4.7. System Preparation and Molecular Docking

The amino acid sequences of hOAT1 and hOAT3 were obtained from the UniProt database (https://www.uniprot.org/, accessed on 22 October 2023) using UniProtKB Q4U2R8 and Q8TCC7, respectively. In addition, the X-ray structure of human CYP2C19 was downloaded from the RCSB Protein Data Bank (PDB ID: 4GQS). The homological models of hOAT1 and hOAT3 were constructed using the SWISS-MODEL web server [28] based on the cryo-EM structure of rat OAT1 bound to *para*-aminohippuric acid (PAH) as a template (PDB ID: 8SDY [21]). The protonation states of all ionizable residues were considered at pH 7.4 using the H++ web server [29]. The SMILES format for PAH, estrone sulfate, and pinocembrin were retrieved from the PubChem database (https://pubchem.ncbi.nlm.nih.gov, accessed on 22 October 2023). The Online SMILES Translator and Structure File Generator (https://cactus.nci.nih.gov/translate, accessed on 22 October 2023) were then used to convert the SMILES strings of each compound into the PDB format. The Gaussian 09 software (Gaussian, Inc., Wallingford, CT, USA) optimized all compounds at the B3LYP/6-31G(d) level. Subsequently, the proteins and ligands were prepared in PDBQT format using AutoDockFR 1.0 [30], and docking was performed with AutoDock Vina 1.2.5 [31]. The dimensions of the grid box were set at 30 × 30 × 30 Å for hOAT1 and hOAT3 and 20 × 20 × 20 Å for human CYP2C19. The grid center coordinates were established as follows: x = 65.06, y = 63.40, and z = 70.68 for hOAT1 and hOAT3; and x = −80.84, y = 22.68, and z = −43.31 for human CYP2C19. The exhaustiveness value was 64, while the remaining parameters were retained at the program’s default settings. The best docked pose of the compounds in the hOAT1, hOAT3, and human CYP2C19 binding sites (i.e., the pose with the lowest Autodock Vina score) was selected for 3D visualization using the UCSF ChimeraX 1.6.1 program [32]. Meanwhile, the 2D interaction diagrams of the protein–ligand complexes were analyzed using the Discovery Studio Visualizer (BIOVIA, San Diego, CA, USA).

### 4.8. Cell-Free CYP450 Inhibition Assay

The CYP450 inhibitory activity of pinocembrin was determined using a P450 screening kit (Life Technologies; Carlsbad, CA, USA). Varied concentrations of pinocembrin were prepared in methanol. Briefly, 40 μL of pinocembrin or positive CYP inhibitor control (miconazole (30 μM) for CYP2B6 and CYP2C19, sulfaphenazole (30 μM) for CYP2C9, or methanol (solvent control) was added to each well of the 96-well black plates. After that, 50 μL of Pre-Mix solution (containing CYP450 BACULOSOMES reagent and regeneration system and reaction buffer) was added to the same 96-well black plates and incubated for 10 min at room temperature (25 °C). A 10 μL mixture of NADP^+^ and substrate was added to each well to start the reaction. Fluorescent changes were monitored by the immediate reading of the plate on the microplate reader (EnVision XCite 2105 Multimode Plate Reader, Packard BioScience, Wellesley, MA, USA). The excitation and emission wavelengths of CYP2B6 and CYP2C19 were 415 and 460, while those of CYP2C9 were 490 and 520, respectively. The fluorescence intensities were monitored every minute for 60 min at room temperature. The percentage of inhibition was calculated according to the following equation:

$$\%\;\mathrm{Inhibition}=(1-\frac{X-B}{A-B})\times 100\%$$

where X is the fluorescence intensity observed in the presence of pinocembrin. A and B are the fluorescence intensity observed in the absence of an inhibitor (solvent control) and the presence of the positive inhibition control, respectively.

### 4.9. Cell Viability Assay

The confluent cells in 96-well plates were treated with pinocembrin for 48 h at 37 °C. After that, the cells were incubated with 0.5 mg/mL MTT solution for four hours. The MTT solution was removed, and DMSO (100 µL) was added to dissolve the crystal formazan. The formazan content was detected at a wavelength of 570 nm using a microplate reader. Cell viability was reported as a percentage of the vehicle-treated cells.

### 4.10. Data Analysis

The results were reported as the mean ± standard deviation (S.D.). The statistical analysis was determined using one-way ANOVA followed by the Tukey test. Statistical significances were considered at a *p* value less than 0.05.

## 5. Conclusions

The present study demonstrates that pinocembrin is a potent inhibitor of hOAT1 and hOAT3 but not of hOCT1, hOCT2, hMATE1, hMATE2, P-gp, and BCRP. It alters CYP2C19 activity and expression. Therefore, the potential hOAT1- and hOAT3- and CYP2C19-mediated drug interaction risks associated with pinocembrin may require attention. To be confident that pinocembrin possesses a clinically relevant drug interaction, further investigation on the pharmacokinetic drug interaction in vivo is needed.

## Figures and Tables

**Table 1:** Summary of IC_50_, Ki, and kinetic parameters of pinocembrin on hOAT1- and hOAT3-mediated 6-CF uptake.

Transporter	IC_50_ (μM)	Ki (μM)	K_m_ (μM)		V_max_ (nmol/min/cm^2^)	
			No Pinocembrin	With Pinocembrin	No Pinocembrin	With Pinocembrin
hOAT1	1.47 ± 0.87	0.81 ± 0.12	12.33 ± 1.32	42.55 ± 2.98 *	5.43 ± 0.25	6.03 ± 1.02
hOAT3	2.38 ± 0.98	1.30 ± 0.24	11.95 ± 1.58	30.20 ± 3.21 *	9.88 ± 1.05	11.32 ± 2.33

Data are shown as mean ± S.D. from three experiments. * *p* < 0.05 compared with the no pinocembrin.

## Data Availability

The data will be made available on request.
